# Overview of Recent Advances in the Design of Plasmonic Fiber-Optic Biosensors

**DOI:** 10.3390/bios10070077

**Published:** 2020-07-09

**Authors:** Yashar Esfahani Monfared

**Affiliations:** Department of Chemistry, Dalhousie University, Halifax, NS B3H 4R2, Canada; y.monfared@dal.ca

**Keywords:** plasmonics, fiber-optic biosensors, photonic crystal fibers

## Abstract

Plasmonic fiber-optic biosensors combine the flexibility and compactness of optical fibers and high sensitivity of nanomaterials to their surrounding medium, to detect biological species such as cells, proteins, and DNA. Due to their small size, accuracy, low cost, and possibility of remote and distributed sensing, plasmonic fiber-optic biosensors are promising alternatives to traditional methods for biomolecule detection, and can result in significant advances in clinical diagnostics, drug discovery, food process control, disease, and environmental monitoring. In this review article, we overview the key plasmonic fiber-optic biosensing design concepts, including geometries based on conventional optical fibers like unclad, side-polished, tapered, and U-shaped fiber designs, and geometries based on specialty optical fibers, such as photonic crystal fibers and tilted fiber Bragg gratings. The review will be of benefit to both engineers in the field of optical fiber technology and scientists in the fields of biosensing.

## 1. Introduction

Since the remarkable revolution in fiber optic technology in 1970s, optical fibers evolved from an optical transmission waveguide to a more complex device for different applications ranging from environmental monitoring to medical imaging [1,2,3]. In particular, optical fiber sensors have been widely used for monitoring temperature [4], pressure [5], mechanical strain [6], concentration of chemical species [7], and refractive index of unknown analytes [8]. The most important type of optical fiber sensors were interferometric-based sensors (based on Sagnac interferometer [9], Mach-Zehnder interferometer [10], or Michelson interferometer [11]), fiber grating sensors [12], and scattering-based fiber optic sensors (based on Rayleigh scattering [13], Brillouin scattering [14], or Raman scattering [15]). While these devices can offer spectacular performances, the cost of these sensing devices on the one hand, and the possible complexity/limited sensitivity of these devices on the other hand, renders them not practical in some applications, especially the industrial, clinical, and commercial large-scale applications which require low production costs and high sensitivity [16]. In the last few years, a new class of fiber-optic sensors based on absorption/intensity measurements has been added to the family of fiber-optic sensors which is called plasmonic fiber-optic sensors.

Plasmonics is one of the fastest growing fields of research, which connected scientists with different backgrounds ranging from chemistry/physics to medical sciences [17,18]. Plasmonics examine the interaction between photons (light) and surface electrons (matter) at the nanoscale. Incoming photons from a light source can excite surface electrons to oscillate collectively, which is called surface plasmons (SPs). Excitation of SPs can result in propagation of surface waves at the intersection of a metal and dielectric nanofilms (surface plasmon polariton or SPP) or can cause a strong localized oscillation of surface electrons in nanoparticles and nanostructures (localized surface plasmon resonance or LSPR) [17,18]. As LSPR and SPP can both cause energy transfer (strong light absorption/scattering) between light and matter at the intersection of nanomaterials, they can be used for sensing and biosensing applications [19].

The most common form of plasmonic biosensors are prism-based surface plasmon resonance (SPR) sensors, which are usually based on Kretschmann configuration [19]. Prism-based SPR sensors are widely used in chemical and biological applications for real-time detection of unknown analytes as highly accurate biosensors [19,20]. However, the most important problem associated with the use of prism-based SPR sensors is bulkiness of the setup, which significantly reduces its applicability in in-situ biosensing, especially biosensing in hard-to-reach locations [18,21]. However, the intersection of plasmonics (SPR sensors) and fiber-optics provide exciting opportunities for scientists working in the fields of biology, chemistry, and medicine to realize SPR sensors in a miniaturized structure [18,21]. This miniaturized structures not only can reduce the size of sensor significantly, but also can offer far more flexibility in terms of the design, materials, and sensing performances of the device.

Plasmonic fiber-optic sensors can solve inherent issues of conventional SPR sensors and add potential new characteristics such as harsh environment tolerance, remote sensing, and distributed sensing to such sensors [18,21]. In recent years, several fiber optic plasmonic biosensing configurations are proposed to detect biological samples and molecules. As different metals can induce different plasmonic properties, optical fibers with different plasmonic materials have also been studied in recent years. While noble metals including gold (Au) and silver (Ag) nanofilms and nanoparticles (NPs) are dominant plasmonic materials in these studies, the field is now experience a rise of alternative plasmonic materials [22] in the design of fiber-optic biosensors such as transition metal nitrides [21], graphene [23], and transition metal oxides [24]. In this review paper, the new advances in the field of plasmonic fiber-optic biosensors, in particular new design concepts and materials, will be discussed in detail.

## 2. State-of-the-Art Plasmonic Fiber-Optic Biosensors Based on Conventional Optical Fibers

**Figure 1:**
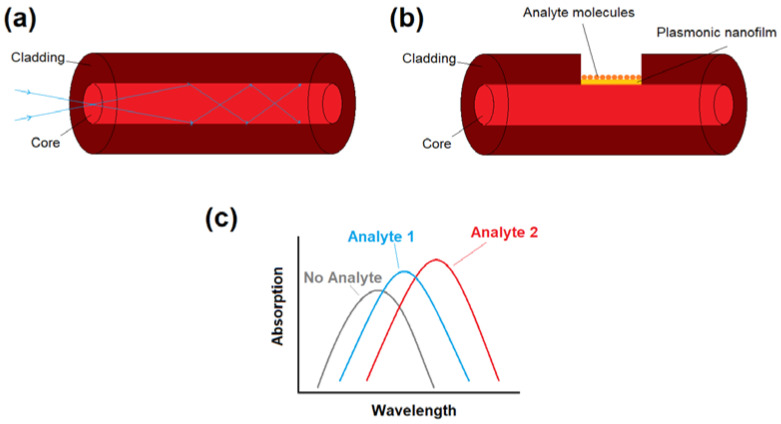
(**a**) Diagram of a conventional optical fiber with a total internal reflection light guiding mechanism. (**b**) Plasmonic fiber-optic biosensor can be obtained by removing a part of the optical fiber cladding and the deposition of plasmonic nanoparticles/nanofilms that are in direct contact with thr biological sample (analyte). (**c**) Variations in absorption spectra (transmission spectra) of light depending on the analyte chemical/physical characteristics. The location of absorption maxima (transmission dips) can be used to detect unknown analytes or monitor the variations in analyte physical/chemical properties.

Conventional optical fibers can enable single mode low loss light transmission with a moderate level of dispersion; therefore, they have been used extensively for fiber-optic telecommunications [25]. Conventional optical fibers are usually made from silica glass and consist of a solid core with the index of refraction n_core_ encircled by a cladding material with slightly lower index of refraction n_clad_ < n_core_ [22]. The incoming light can be confined in the core region of a conventional optical fiber if the incidence angle is smaller than a critical angle [25]. This mechanism of light confinement in conventional optical fibers is called total internal reflection [25], which is illustrated in the Figure 1a.

To use conventional fibers for plasmonic biosensing, fiber geometries can be modified to trap the light inside the core, which is in direct contact with the plasmonic material. For example, all or part of the cladding of the fiber is removed (for instance by chemical itching or side polishing) for the biosensing applications, as sketched in Figure 1b. Plasmonic nanoparticles or nanofilms are also in direct contact with analyte molecules. Either the energy exchange and interaction between fiber mode in the core, and plasmonic modes on the surface of nanofilms/nanoparticles, or the variations in absorption spectra of nanoparticle solution itself, can induce transmission dip (or absorption peak) in the light transmission spectra depending on the analyte characteristics (for instance refractive index). As each molecule has a unique refractive index (RI), each analyte can induce different absorption peaks and therefore, the proposed structure can act a biosensor by monitoring the variations in absorption maxima, as demonstrated in Figure 1c. The main geometries of plasmonic fiber-optics based on conventional optical fibers includes unclad fibers, side-polished (or D-shaped) fibers, tapered, and U-shaped fibers, which will be reviewed in detail in the next subsections.

### 2.1. Unclad (or Exposed Core) Plasmonic Fiber-Optic Biosensors

**Figure 2:**
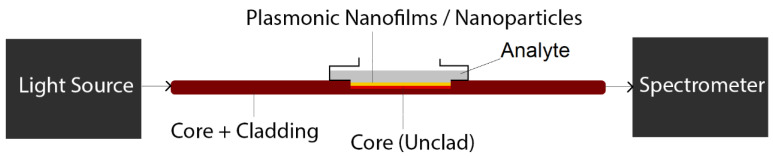
The schematic of a potential plasmonic biosensing setup based on unclad (exposed core) optical fibers.

The most straightforward configuration for conventional optical fiber-based plasmonic biosensing consist of an optical fiber, which has a partial or entirely naked core by removing the fiber cladding using for example a sharp blade [26,27,28] (followed by a cleaning procedure with de-ionized water and acetone). To facilitate the process of cladding removal, polymer or plastic based fiber-optics are the desired type of fiber for this type of biosensor [26,27,28]. The plasmonic nanoparticles/nanofilms can be then deposited on the surface of unclad section of fiber. Then, biomolecules can be attached to plasmonic nanoparticles or nanofilm surfaces at the final stage [26,27,28]. The schematic of such sensors and the required setup for biosensing is demonstrated in Figure 2. Note that depending on the setup the biological sample (analyte) can be a solution in contact with nanofilms (such as Figure 2) or it can be molecules attached to nanoparticles.

As different biological samples/molecules induce different plasmonic responses, either the absorption spectra of light transmission in fiber or absorption spectra of nanoparticles can be used to detect an unknown analyte or monitor the changes in biological sample. There were quite a few scientists and research groups that have worked on developing unclad plasmonic fiber-optic biosensors in recent years.

**Figure 3:**
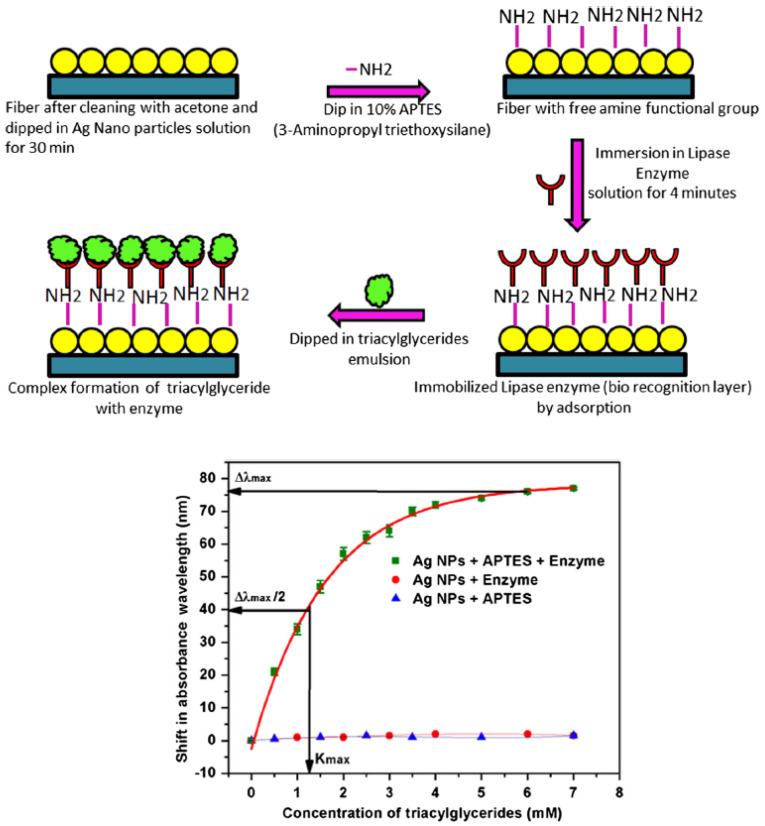
(top panel) The schematic of the process of lipase enzyme immobilization to create the biorecognition surface on the plasmonic fiber-optic sensor. (bottom panel) Peak absorbance wavelength shift as a function of triacylglycerides concentration in pH 7.4 solution at 37 °C standard deviation with APTES and enzyme, with APTS only and with enzyme only. Figures reproduced under the terms of the CC-BY Creative Commons attribution 4.0 from Reference [26], Copyright 2017.

In 2017, Baliyan et al. [26] reported a fiber-optic SPR-based sensor for detection of triacylglycerides by immobilizing lipase enzyme on silver nanoparticles (Ag-NPs) coated on an unclad segment of a conventional optical fiber. A 15 cm plastic-clad silica optical fiber was used as a fiber-optic with a 600 μm core diameter [26]. The main reason for choosing a plastic clad fiber in this study was ease of removal of the fiber cladding from a 1 cm middle section of the fiber length [26]. To deposit NPs, the fiber probe was coated by using a dip coating method. In this method, the fiber was dipped vertically into a long narrow cylindrical vessel containing the Ag-NPs solution [26]. To functionalize the probe, plasmonic-coated fiber was dipped in 10% ethanolic solution of 3-Aminopropyl triethoxysilane (3-APTES) for two minutes to create the amino functional group on the surface of NPs. Finally, the functionalized optical fiber probe was immersed into lipase enzyme solution which can be immobilized by the interaction with amine groups of APTES on the surface of plasmonic-coated fiber [26]. The schematic of the immobilization method proposed by Baliyan et al. [26] to create the biorecognition layer on the fiber probe is demonstrated in Figure 3 (top panel). For the characterization of the probe, different concentrations of triacylglyceride solution, ranging from 0 to 7 mM, were poured into the flow cell one by one, and the absorbance spectrum was recorded by the spectrometer. Using the plasmonic fiber-optic probe, a sensitivity of 28.5 nm/mM of triacylglyceride solution is obtained with a limit of detection of 0.016 mM in the entire range, as demonstrated in Figure 3 (bottom panel) [26].

As another example, Mishra et al. [27] used a fiber optic SPR-based sensor for detecting of CrO_4_ in solutions by coating layers of silver, indium-tin oxide (ITO), and hydrogel over the unclad core of an optical fiber. To prepare the fiber-optic probe, about 17 cm of a conventional optical fiber was used, where the cladding from the middle portion (about 1 cm in length) was removed using a sharp blade [27]. After cleaning with deionized water, acetone, and high-tension ion bombardment in a vacuum chamber, a 40 nm thick nanofilm of Ag and a 7 nm thick nanofilm of ITO were deposited over the unclad portion of fiber [27]. To create bonds with the hydrogel and also improve the adhesion of the hydrogel, the silanization process was performed by inserting the cleaned surface of the probe into a solution of trimethoxysilane prepared in Millipore water. Finally, hydrogel coating over the plasmonic nanofilms was achieved using a dip coating method [27]. Mishra et al. [27] reported a sensitivity of 6.67 × 10^10^ nm/M and a limit of detection of 0.5 × 10^−11^ M using this plasmonic fiber-optic probe.

**Figure 4:**
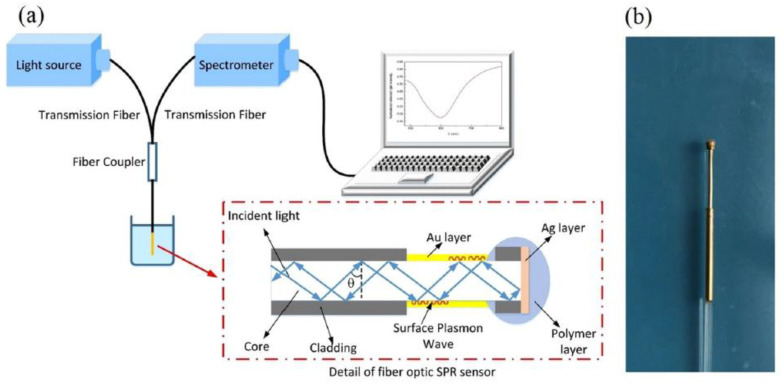
Plasmonic fiber optic sensing system in [28], (**a**) schematic of the experimental setup including light source, transmission fibers, plasmonic fiber-optic sensor, and spectrometer and (**b**) the actual photograph of the fabricated plasmonic fiber optic sensor. Figures reproduced under the terms of the CC-BY Creative Commons attribution 4.0 from Reference [28], Copyright 2017.

Unclad plasmonic fiber-optic biosensors also used for detection proteins. For instance, Wang et al. [28] designed a fiber-optic plasmonic biosensor with a 50 nm gold layer deposited on the unclad part of a conventional optical fiber, for the detection of the C-reactive protein (CRP), as illustrated in Figure 4.

**Figure 5:**
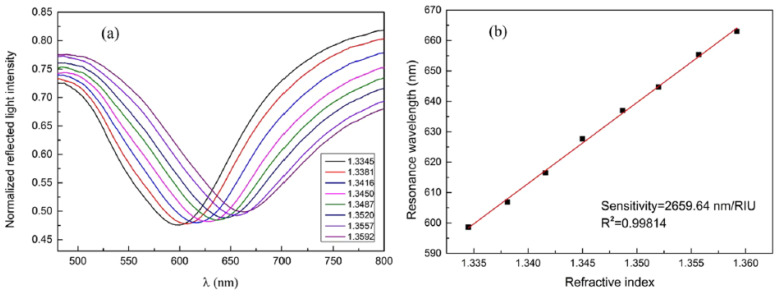
(**a**) Reflection spectra (normalized) of NaCl solutions with different refractive indices (RIs). (**b**) Experimental relationship between resonance wavelength location and RI of the solution. Figures reproduced under the terms of the CC-BY Creative Commons attribution 4.0 from Reference [28], Copyright 2017.

Figure 5a shows normalized reflection spectra of the fiber optic SPR sensor in measuring NaCl solutions with different concentrations. The SPR dip shifts towards longer wavelengths with the increase of RI, and the response of the resonance wavelength shows a linear relation with the RI change, as shown in Figure 5b. The sensitivity of the sensor is 2659.64 nm/RIU for RI ranging from 1.3345 to 1.3592, which is sufficiently high for biosensing purposes [28]. For protein detection application, they show that the fiber has a linear response to CRP concentrations within the range from 0.01 to 20 μg/mL. They reported a shift of 1.17 nm per lg (μg/mL), for resonance wavelength per unit change in the logarithm concentration of CRP [28].

### 2.2. Side-Polished (D-Shaped) Plasmonic Fiber-Optic Biosensors

**Figure 6:**
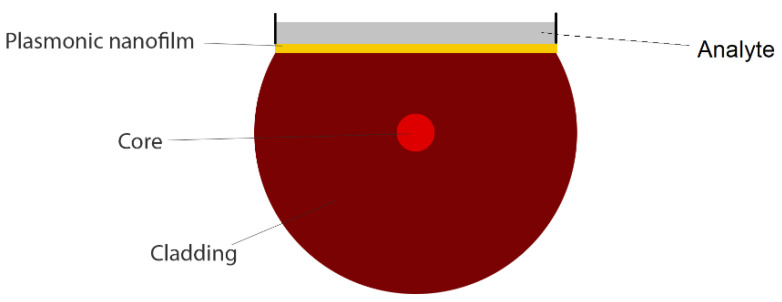
Cross section of a side-polished (D-shaped) plasmonic fiber-optic biosensor for liquid analyte detection.

Side polishing the cladding section of a conventional optical fiber is another method to design a plasmonic fiber-optic biosensor. In these geometries, the cladding section of fiber will be polished (via, for example, the wheel polishing technique [29]), and then plasmonic nanofilms or nanoparticles will be deposited on the flat surface of fiber (via, for example, the vacuum evaporating method [29]), as illustrated in Figure 6. The interaction between fiber and plasmonic modes can form a transmission dip (absorption peak) in light transmission spectra, depending on the biological sample refractive index (RI) on the surface of nanofilms/nanoparticles [29,30,31]. This mechanism can be used to detect an unknown molecule or monitor the variations in analyte RI.

In 2019, Dong et al. [29] experimentally studied a side-polished few-mode fiber (FMF) SPR-based biosensor using a gold thin film. They reported a RI detection range of 1.333 to 1.404, and as an example, they demonstrated the biosensing performance of their sensor by testing bovine serum albumin (BSA) solution. They reported an averaged BSA RI sensitivity of 6328 nm/RIU and an averaged BSA concentration sensitivity of 1.17 nm/(mg/mL) [29].

**Figure 7:**
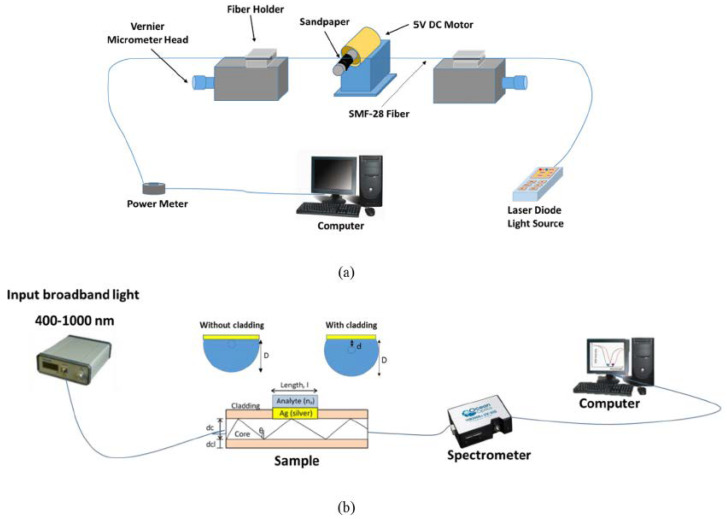
(**a**) Schematic demonstration of fabrication process of side-polished optical fiber and (**b**) experimental setup of the D-shaped plasmonic fiber-optic sensor including broadband light source and spectrometer. Figures reproduced under the terms of the CC-BY Creative Commons attribution 4.0 from Reference [31], Copyright 2019.

In 2019, Zainudding et al. [30] theoretically, numerically, and experimentally proposed an SPR-based optical fiber side-polished refractive index biosensor using a silver nanofilm. The side-polished optical fiber device was fabricated using the wheel polishing method; a schematic diagram of this method is demonstrated in Figure 7a. To polish the side of the fiber, a 2 cm of coating of a Corning SMF-28 optical fiber was first removed to expose the glass part of the fiber (core and cladding) [30]. The fiber was then fastened at either end with fiber clampers [30] and a sandpaper grinding wheel was used to reduce the thickness of the fiber by varying the speed of the wheel rotation using a computer program, as demonstrated in Figure 7a [30]. The D-shaped fiber probe was finally coated with a 40 nm silver nanofilm deposited on top of the fiber core [30]. The experimental setup in their study consist of a broadband light source having a range from 400 to 1000 nm, side-polished optical fiber, and a spectrometer to detect the light, as illustrated in Figure 7b [30]. Using the mentioned side-polished fiber designs, they reported a sensitivity of 2166 nm/RIU and 208.333 nm/RIU to distilled water (*n* = 1.333) and alcohol (*n* = 1.345), respectively.

Furthermore, in 2018, Melo et al. [31] numerically proposed a D-shaped plasmonic fiber optic biosensor with plastic as main materials and graphene on silver as coating materials with a peak sensitivity of 5161 nm/RIU. They discussed the effect of polishing depth and sensing area length on the performance of the biosensor [31]. The conclusions from this study reveals that the higher polishing depth provides the narrower curves for normalized transmitted power, and smaller sensing area lengths leads to better quality parameters, regardless of the number of graphene layers [31].

### 2.3. Tapered Plasmonic Fiber-Optic Biosensors

**Figure 8:**
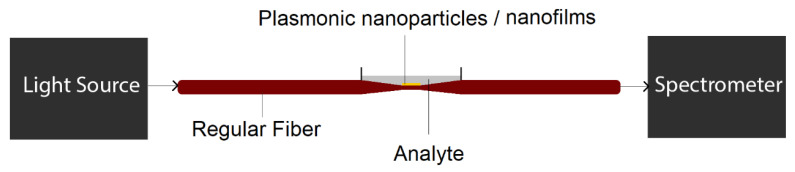
The schematic of a potential plasmonic biosensing setup based on tapered optical fibers.

Tapered fibers are much simpler devices compared to side polished or unclad plasmonic fiber-optic sensors [32,33]. The tapering process can be achieved via different methods, for example the hydrogen-oxygen flame-brushing technique [32]. In this process, both sides of the fiber are pulled simultaneously while heating the fiber to its softening temperature, which result in tapering the fiber geometry [32]. In tapered optical fibers, the reduction of the core and cladding diameters due to tapering process makes the evanescent fields spread out into the cladding region and even surface of cladding [32]. As a result, tapered plasmonic fiber-optic sensors can be realized as a sensitive platform for biosensing, as demonstrated in Figure 8. In plasmonic tapered optical fibers, the sensing strategy is usually based on the interrogation of the transmission intensity variations in the evanescent field absorption of deposited plasmonic nanoparticles on the tapered fiber surface.

Lin et al. [32] proposed a tapered SPR-based fiber-optic biosensor for monitoring anti-DNP (N-(2,4-dinitrophenyl)-6-aminohexanoic acid) antibody. The Au nanoparticles were used as plasmonic material and aqueous samples were in contact with the tapered area of fiber directly [32]. The tapered optical fiber in their study was fabricated by tapering a standard SMF-28 fiber through a hydrogen-oxygen flame-brushing technique, as discussed above [32]. The experimental setup of their study consists of a 532 nm laser light source that has been coupled into the fiber to induce the LSPR of immobilized Au nanoparticles. The light beams were modulated by the optical chopper at 500 Hz and was demodulated by a lock-in amplifier to improve the signal-to-noise ratio [32]. The transmitted optical beams for biosensing were monitored in real-time by a photodiode which has been amplified by the lock-in amplifier [32]. They studied anti-DNP antibodies with different concentrations ranging from 5 × 10^−9^ to 1 × 10^−6^ g/mL, and reported a refractive index resolution of 3.2 × 10^−5^ RIU [32].

There is more room for future improvement of the performance of tapered plasmonic fiber-optic biosensors by varying the design parameters of these fibers, for example its tip diameter [33]. For instance, Huang et al. [33] theoretically studied tapered plasmonic biosensor sensitivities and realized that as the tip end diameter decreases, the refractive index sensitivity of tapered sensors can increase dramatically.

### 2.4. U-Shaped Plasmonic Fiber-Optic Biosensors

**Figure 9:**
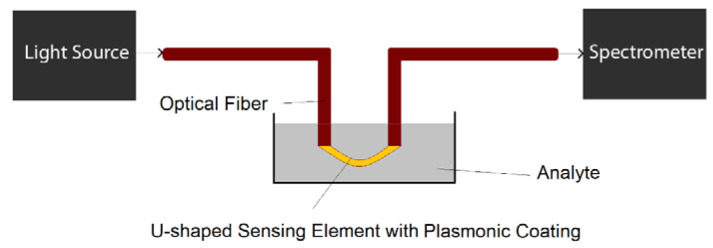
The schematic of a potential biosensing setup based on U-shaped plasmonic optical fibers.

U-shaped plasmonic fiber-optic probes can be prepared by fiber bending with the help of a heat source, for example butane flame [34]. The U-shaped region of fiber is usually de-cladded in order to deposit plasmonic nanoparticles [34] or nanofilms [35] on the surface of fiber-optic probe. The U-shaped region is usually in direct contact with analyte to detect or track the changes in analyte physical/chemical properties, as illustrated in Figure 9.

In 2019, Shukla et al. [34] reported a U-shaped fiber optic probe coated with glucose capped Ag nanoparticles for the detection of mercury ions in aqueous solution. U-shaped optical fiber probes were prepared by removing 1 cm length of the cladding region (in the center of the fiber) followed by bending the optical fiber using a butane flame [34]. The U-region was cleaned using the standard acid-alkali protocol after fabrication, and the probe was dipped in glucose capped Ag NPs solution [34]. Finally, the glucose capped Ag NPs coated optical fiber probe was dipped in mercury solution of different concentrations for biosensing application [34]. The reaction between Ag NPs and mercury ions used as the sensing mechanism as the plasmonic absorbance reduces following this reaction [34]. The detection limit of the proposed sensor reported to be as low as 2 ppb for mercury detection.

**Figure 10:**
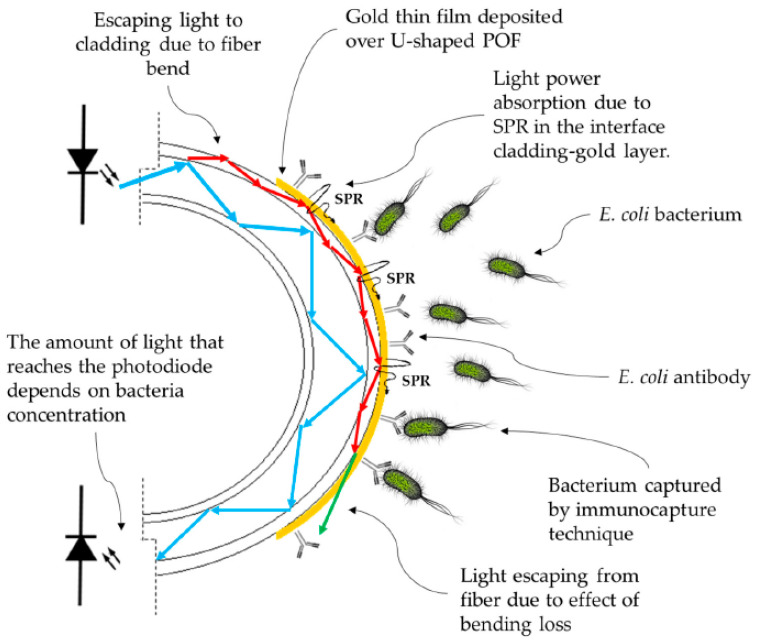
The working principle of gold-coated U-shaped plasmonic plastics-based optical fiber (POF) biosensor for detection of Escherichia coli (*E. coli*) bacteria. Figure reproduced under the terms of the CC-BY Creative Commons attribution 4.0 from Reference [35], Copyright 2018.

As another example, Arcas et al. [35] proposed a gold-coated U-shaped plastic optical fiber biosensor for Escherichia coli (*E. coli*) bacteria detection in 2018. While bacterium presents a RI about 1.39 at 400–800 nm, which is slightly higher than that of pure water (1.333), the bacteria concentration in real-world applications is not high enough to substantially change the RI of the water [35]. Therefore, one should concentrate the target bacteria (*E. coli* in this case) around the fiber via different techniques, for example immunocapture [35], which includes bonding the specific antibody on the sensing region in a way that only bacteria of specific species are captured and fixed on the sensor surface. The working principle of their U-shaped optical fiber biosensor was based on fiber bending and bending loss. As an increase in RI in the surrounding medium of a U-shaped fiber optic increases the critical angle of the cladding interface at U-region, this can lead to an increase in bending loss of the fiber and an attenuate peak in transmission spectra of light in the fiber. The dependence of light intensity on bacteria concentration in a U-shaped plasmonic fiber-optic biosensor is schematically demonstrated in Figure 10.

**Figure 11:**
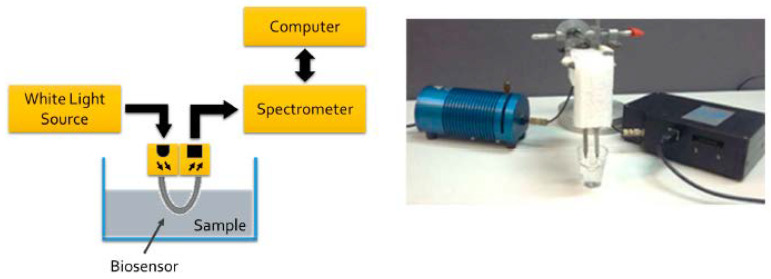
The schematic overview of experimental setup of U-shaped plasmonic fiber-optic biosensor with a white light source (left panel) and the actual setup with light source on the left and spectrometer on the right side of the fiber probe (right panel). Figure reproduced under the terms of the CC-BY Creative Commons attribution 4.0 from Reference [35], Copyright 2018.

The authors used two different setups (with two different light sources, white light source 350–845 nm and 880 nm LED source) in this investigation [35]. Their second setup (including the white light source) is illustrated in Figure 11. Using the U-shaped plasmonic fiber-optic biosensor, the authors reported a detection limit of 1.5 × 10^3^ colony-forming units (CFU)/mL for their proposed biosensor [35]. They also compared the efficiency of two setups for bacteria detection. Their second setup (with white light source) was capable to detect up to 10^3^ CFU/mL, whereas Setup 1 (with LED at 880 nm) was able to the detect up to 10^6^ CFU/mL.

## 3. State-of-the-Art Plasmonic Biosensors Based on Specialty Optical Fibers

The conventional optical fibers impose two major limitations for the design of biosensors. First, the index of refraction deviations of the core and cladding are restricted due to a limited fiber geometry. Second, one cannot use a low index/high index material in the core/cladding of the optical fiber due to a breakdown of total internal reflection guiding mechanism. Therefore, plasmonic fiber-optic biosensors based on specialty optical fibers have been proposed and investigated in the literature as alternatives to conventional optical fiber-based biosensors with more flexibility in terms of the design and characteristics. The most important specialty fibers for plasmonic-based biosensing are photonic crystal fibers, and tilted fiber gratings.

### 3.1. Plasmonic Biosensors Based on Photonic Crystal Fibers

**Figure 12:**
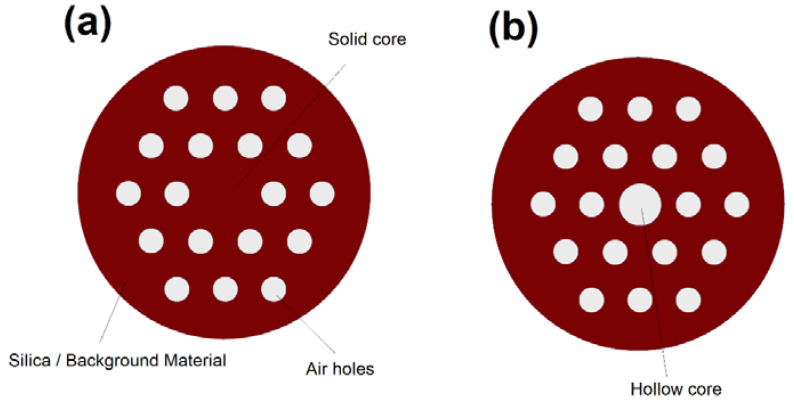
Cross section of a photonic crystal fibers (PCF) with a (**a**) solid core and (**b**) hollow-core. Note that PCF has several design parameters that can be adjusted to obtain the desired optical properties. The design parameters include core size, air-holes dimension, background material, center-to-center distance between air holes, and number of air holes.

Photonic crystal fibers (PCFs) are a flexible and adjustable class of optical fibers that open the door to new possibilities in nonlinear optics [36], optical sensing [37], and optical imaging [38]. As demonstrated in Figure 12, PCFs have a core that is encircled by a periodic array of air-holes that works as a cladding of the fiber [11]. PCFs offer numerous unique features such as the possibility of realizing ultra-flattened dispersion, endlessly single mode operation, low propagation losses, and controllable nonlinearity [36,37,38].

We can divide PCFs to two major categories based on their design: solid-core PCFs [36] and hollow-core PCFs [39]. Similar to conventional optical fibers, solid-core PCFs operate based on TIR mechanism [36]. In hollow-core PCFs (HCPCFs) the guiding mechanism strongly depends on the core material [36]. TIR can be the guiding mechanism in HCPCFs if one fill the core of the fiber with a high index material such as a high index liquid [36]. However, total internal reflection is not possible if we fill the core with a low index material such as a gas [39]. Interestingly, the light guidance in hollow-core PCFs filled with low-index material can still be possible via a photonic bandgap mechanism over a narrow bandwidth [39].

For plasmonic biosensing applications, both solid-core and hollow-core PCFs can be utilized as host mediums to plasmonic materials and analyte. For example, liquid analytes can be filled into the hollow-core of PCFs for high refractive index biosensing applications [36]. The most important plasmonic biosensors based on PCFs are D-shaped PCF biosensors, but the other possible design concepts based on PCFs will also be described in detail in the next subsections.

#### 3.1.1. D-Shaped PCF-Based Plasmonic Biosensors

**Figure 13:**
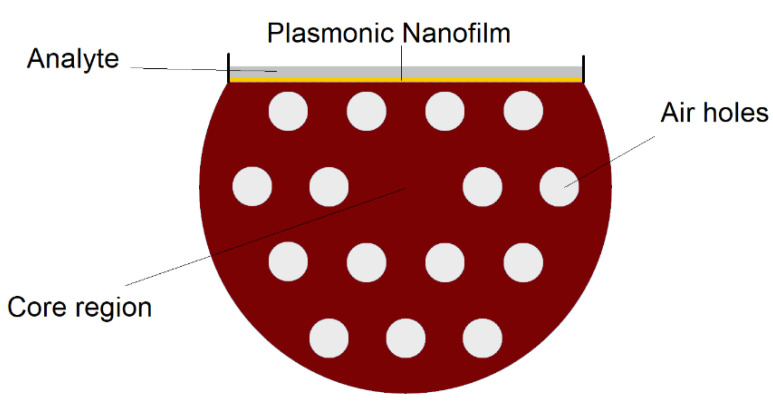
Cross section of D-shaped PCF-surface plasmon resonance (SPR) biosensor that can be used to detect analytes with low or high refractive indices, depending on the design parameters.

In D-shaped PCF plasmonic (or SPR based) biosensors, the interaction between fiber mode at the core of the fiber and the plasmonic modes at the surface of nanofilms/nanoparticles depending on the analyte RI can be used to detect an unknown analyte (or the change in analyte physical/chemical characteristics) [37,40]. The air holes of PCF can help with providing flexibility for phase matching (index matching) between fiber mode and plasmonic mode [37,40]. D-shaped PCF biosensors are one of the most popular design concepts in RI sensing as it can provide a great platform for using different plasmonic materials, and detection of analytes with low or high RIs [37,41]. The cross-sectional view of a D-shaped PCF-based plasmonic biosensor is illustrated in Figure 13.

In 2018, Lu et al. [40] presented a high-resolution D-shaped PCF-SPR biosensor based on gold gratings. They reported a maximum resolution of 5.98 × 10^−6^ RIU in the RI range of 1.36–1.38, and spectral sensitivity of 3340 nm/RIU [40]. Liu et al. [41] reported a symmetrical dual D-shaped PCF-SPR sensor with a RI detection range between 1.36 and 1.41 and an average spectral sensitivity of 14,660 nm/RIU, which was significantly higher than previously reported D-shaped PCF-SPR biosensors.

**Figure 14:**
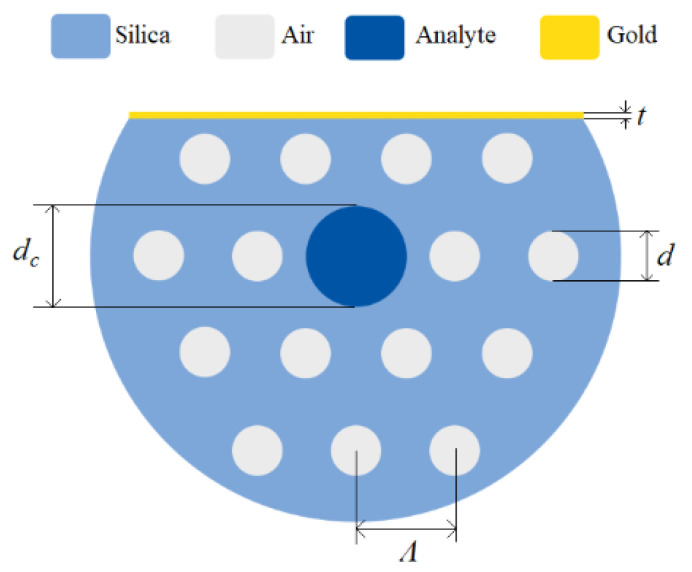
Cross section of the D-shaped PCF-based plasmonic biosensor in [37] with air holes diameter *d*, analyte-core diameter *d_c_*, holes pitch *Ʌ*, and gold layer thickness *t*. Reproduced with permission from Reference [37], Copyright 2019, IEEE.

In 2019, Monfared et al. [37] proposed a D-shaped PCF-based plasmonic biosensor using gold nanofilm for high-index analyte detection. The main novelty of this work was focusing the plasmonic fiber-optic biosensors on high index liquid analyte detection, as most of the biosensing structures can only detect analytes with an RI less than 1.44, which is the refractive index of fiber core [37]. If the RI of analyte (which is usually located around the fiber) is larger than 1.44, the TIR guiding mechanism of fiber cannot be satisfied, and therefore, there is no guiding mode in the fiber core [37]. However, they filled the PCF core with analyte to satisfy the TIR mechanism even with high index liquid analytes with RI between 1.45 and 1.6 [37]. The schematic of their proposed PCF-based plasmonic fiber-optic sensor with a gold nanofilm on the polished side of the PCF is illustrated in Figure 14.

**Figure 15:**
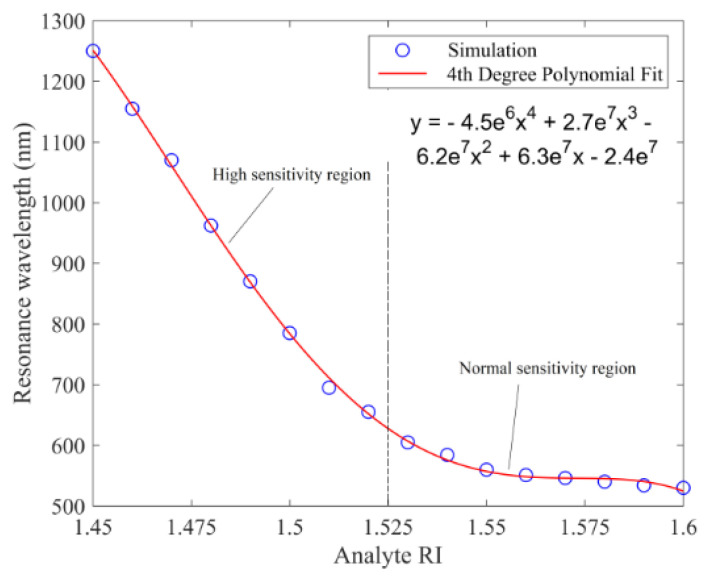
Loss peak wavelength or resonance wavelength as a function of analyte RI for a D-shaped PCF-based plasmonic biosensor. Reproduced with permission from Reference [37], Copyright 2019, IEEE.

Their proposed sensor has a RI detection range from 1.45 to 1.6, and exhibit linear sensing performance with a RI spectral sensitivity of 9300 nm/RIU for analyte RI ranging from 1.45 to 1.525 (which is considered a high sensitivity region), and 1176 nm/RIU for analyte RI ranging from 1.525 to 1.6 (which is considered a normal sensitivity region of the sensor) [37]. These sensing regions are demonstrated in Figure 15. They also reported maximum amplitude sensitivities of 183.6 RIU^−1^ for 785 nm excitation and 820 RIU^−1^ for 1050 nm excitation [37].

**Figure 16:**
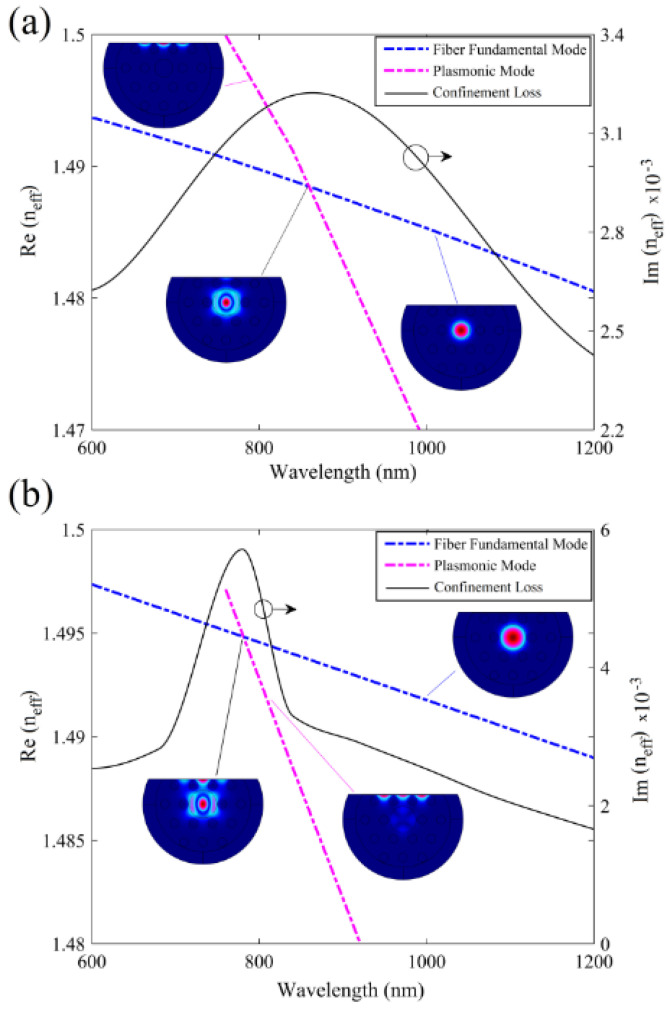
Real and imaginary parts of effective index of fiber mode and plasmonic mode in a D-shaped plasmonic fiber-optic biosensor as a function of wavelength in (**a**) with a core size of *d_c_/Ʌ* = 0.46 and (**b**) with a core size of *d_c_/Ʌ* = 0.7. The other design parameters are n_analyte_ = 1.5, *d/Ʌ* = 0.23, *Ʌ* = 6 μm and *t* = 50 nm. Insets show field distribution at different points in spectra for fiber, plasmonic, and the coupled core-plasmonic modes. Reproduced with permission from Reference [37], Copyright 2019, IEEE.

The sensing mechanism of their proposed biosensor was based on interaction between fiber mode in the core and plasmonic mode on the surface of gold nanofilm, as demonstrated in Figure 16 [37]. As illustrated in Figure 16a,b, when the effective mode indices of the fiber mode and the plasmonic mode cross over, a loss peak appear that is sensitive to RI of analyte in the fiber core, and can be used to detect an unknown analyte [37]. The results in Figure 16 also demonstrate the role of design parameters such as air-holes dimension, core size, distance between air holes, and thickness of plasmonic nanofilm on the location of loss maxima.

In 2020, Monfared [21] reported a D-shaped PCF-based plasmonic biosensor using titanium nitride as an alternative plasmonic material (plasmonic nanofilm) with a RI detection range of 1.44 to 1.52 and two linear sensing regions with an average spectral sensitivity of −16,275 nm/RIU for analyte RI ranging from 1.44 to 1.48, and −7571 nm/RIU for analyte RI between 1.485 and 1.52, respectively [21]. While the detection range of this biosensor was more limited comparing to [37], the main advantages of this biosensor were utilizing a cost-effective material (titanium nitride) instead of gold, and also showing a higher sensitivity and figure of merit comparing to gold-based D-shaped PCF biosensors.

#### 3.1.2. Other PCF-Based Plasmonic Biosensors

**Figure 17:**
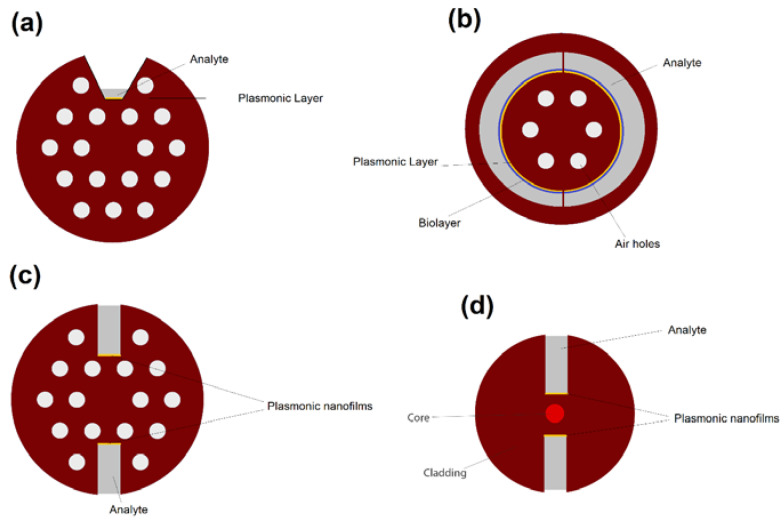
Cross section of PCF-based plasmonic biosensors based on a (**a**) trapezoidal-channel, (**b**) semi-circular channels, (**c**) H-shaped geometry with air holes, and (**d**) H-shaped microstructured geometry.

As PCF is an extremely flexible platform for realizing different design concepts, a major diversity in terms of the possible designs for plasmonic biosensors based on PCFs can be seen in the literature [42,43,44]. In contrast to the common D-shaped and H-Shaped PCFs, in 2019, Chu et al. [42] proposed a six-fold PCF-based plasmonic biosensor with a trapezoidal analyte channel and gold layer for the detection of high RI liquid analytes. They reported sensitivities in terms of liquid analyte heights ratios of 20%, 25%, 30%, and 50% of the maximum channel height, with a maximum RI sensitivity of 4400, 6100, 8000, and 17,000 nm/RIU, respectively, for analyte RI ranges of 1.44–1.57, 1.41–1.51, 1.40–1.49, and 1.40–1.44. A generalized cross-sectional view of the trapezoidal-channel PCF-based biosensors is illustrated in Figure 17a.

As another example, Hassani et al. [43] theoretically proposed a PCF-based biosensor with semi-circular channels containing analyte for detection of biolayer thickness and reported resolutions as high as 0.039–0.044 nm for biolayer thickness in 600–920 nm region. A simplified and generalized cross-sectional view of their proposed biosensor is illustrated in Figure 17b.

**Figure 18:**
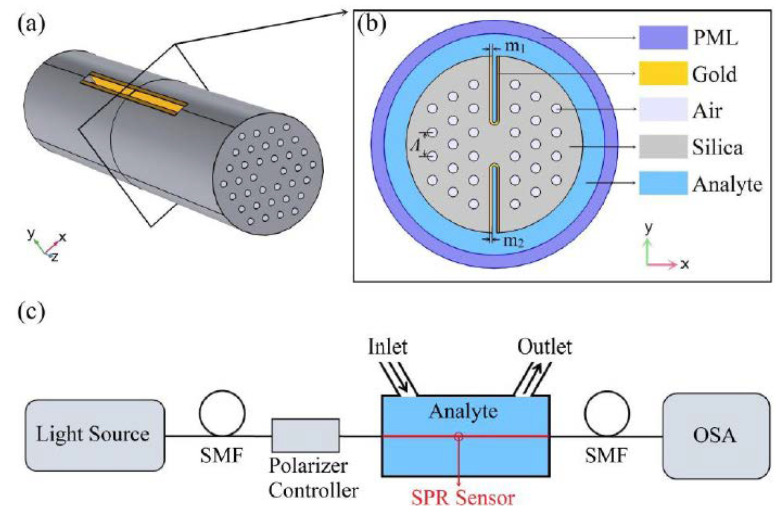
(**a**) Three-dimensional schematic view of the proposed H-shaped photonic crystal fiber-based surface plasmon resonance (PCF-SPR) sensor; (**b**) cross-section of the SPR sensor; (**c**) possible experimental setup of the SPR sensor for analyte RI detection. Figure reproduced under the terms of the CC-BY Creative Commons attribution 4.0 from Reference [44], Copyright 2020.

H-shaped PCFs is another fiber geometry that demonstrated potential for designing highly accurate and ultra-sensitive plasmonic biosensors [44]. The cross-sectional view of plasmonic H-shaped PCF biosensors are demonstrated in Figure 17c. As seen in Figure 17 and Figure 18, the H-shaped geometry consists of a solid (or hollow) core encircled with an array of airholes and two grooves alongside of the core of PCF. The grooves are usually filled with analyte, which is in direct contact with plasmonic nanofilms/nanoparticles.

In 2020, Han et al. [44] theoretically and numerically reported a new design of H-shaped PCF-based SPR sensor for detecting analytes with a large detection range. The possible experimental setup, three-dimensional diagram of the sensor and the cross-sectional view of the fiber probe is illustrated in Figure 18. According to Figure 18, the grooves of their proposed H-shaped PCF as the sensing channels are coated with gold nanofilms and then brought into direct contact with the analyte [44]. Interestingly, the analyte RI in this design can be either higher or lower than that of the fiber materials, which is a definite advantage of this design over the similar plasmonic fiber-optic probes. The H-shaped structure of their proposed fiber probe can be fabricated using femtosecond laser micromachining, focused ion-beam milling, or chemical etching of the original PCF [44].

**Figure 19:**
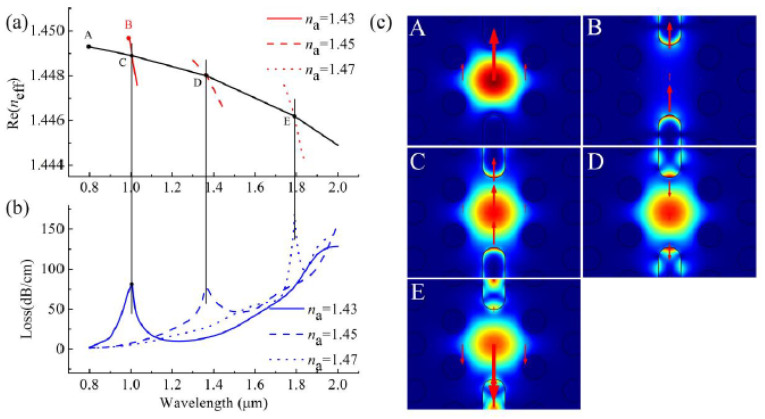
(**a**) Real part of the y-polarized core mode (black solid curve) and y-polarized surface plasmon polariton (SPP) mode at analyte RI of 1.43, 1.45, and 1.47 (red solid, red dashed, red dotted curves); (**b**) Imaginary part or loss spectra of the y-polarized core mode at analyte RI of 1.43, 1.45, and 1.47; (**c**) y-polarized electric field distributions of core mode and SPP mode at different wavelengths (A for core mode at 800 nm, B for SPP mode at 992 nm, C for core mode at 1006 nm with *n*_a_ = 1.43, D for core mode at 1367 nm, and E for core mode at 1791 nm with *n*_a_ = 1.47). Figure reproduced under the terms of the CC-BY Creative Commons attribution 4.0 from Reference [44], Copyright 2020.

They mainly studied the sensing performance of the y-polarized component of the core mode as it can leads to stronger plasmonic coupling between the core and plasmonic modes [44]. In Figure 19, the coupling properties of their proposed H-shaped biosensor as well as index matching between fiber mode and plasmonic modes for different analyte RIs is illustrated. The Figure 19 show that the proposed biosensor has potential for the detection of analytes with higher or lower RI than that of the fiber materials. Using the H-shaped PCF-based plasmonic biosensor, they reported an analyte RI detection range between 1.33 and 1.49, with a maximum sensitivity of 25,900 nm/RIU at an analyte RI range of 1.47–1.48.

Another possibility in biosensor design using specialty optical fibers is open-structured micro-structured optical fiber sensors in which the grooves can easily be coated with metal, where the analyte is in direct contact with the metal surface [45,46]. Micro-structured H-shaped plasmonic biosensors can be geometrically different from PCFs, a cross-sectional view of generic form of these sensors is illustrated in Figure 17d. For example, Erdmanis et al. [45] numerically studied an H-shaped plasmonic micro-structured optical fiber sensor coated with a thin gold layer covered by a uniform titanium dioxide (TiO_2_) layer. With single mode operation in the vicinity of 1550 nm, they reported a theoretical refractive-index resolution of up to 5 × 10^3^ nm/RIU [45]. Gomez-Cardona et al. [46] reported an H-shaped micro-structured optical fiber SPR-based biosensor with symmetrical dielectric–metal–dielectric waveguide. Their proposed sensor has a peak 27 nm of full width at half maximum (FWHM), a high sensitivity of 7540 nm/RIU and figure of merit of 280 RIU^−1^ [46].

### 3.2. Plasmonic Biosensors Based on Tilted Fiber Bragg Gratings

Fiber Bragg gratings (FBGs) can be described as narrow pass/rejection band filters which can be implemented in optical fibers [47]. These gratings can have applications in fiber lasers, dispersion compensators and optical amplifiers [47,48]. As the Bragg wavelength is sensitive to pressure, strain, and temperature, the application of FBGs in sensing (especially mechanical sensing) exceed the other applications of these gratings in optics and photonics [47,48,49].

Tilted fiber Bragg grating (TFBG) is essentially an FBG in which grating planes are angled by a few degrees relative to the fiber axis plane [47,48,49]. The grating tilt will break down the cylindrical symmetry of the fiber which can significantly shift the coupling properties of the overall structure [47,48,49]. The TBFGs provide a strong interaction medium for the modes in the fiber core and cladding, which can enhance the plasmonic sensitivity to the analyte physical/chemical properties [47,48,49].

**Figure 20:**
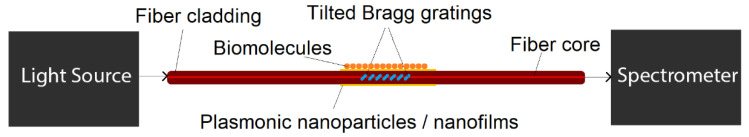
The schematic of a potential plasmonic biosensing setup based on tilted fiber Bragg gratings.

To design a plasmonic biosensor based on TBFGs, one can combine these properties with plasmonic nanofilms or nanoparticles. The most important advantage of this geometry is that one does not need to remove parts of the fiber cladding to access core guided light as gratings can diffract some of the light into the cladding [48,49]. The TBFGs will enhance the mode coupling between core and cladding modes which can then utilized to excite plasmonic modes, the potential setup of such biosensing device is demonstrated in Figure 20.

In 2017, Guo et al. [48] reported a metal-coated plasmonic TFBG imprinted in a commercial single-mode fiber core for biosensing applications. They reported a pM level in lab and nM level in real samples limit of detection, and 500 nm/RIU and 8000 dB/RIU as sensitivities [48]. In 2020, Lobry et al. [49] also reported a plasmonic TBFG biosensor functionalized with aptamers oriented against HER2 (Human Epidermal Growth Factor Receptor-2), a relevant protein biomarker for breast cancer diagnosis. The gold coated multimode TFBG sensors demonstrated a sensitivity of 124.89 nm/RIU, which was an enhancement of 22% compared to single mode TFBGs [49].

**Figure 21:**
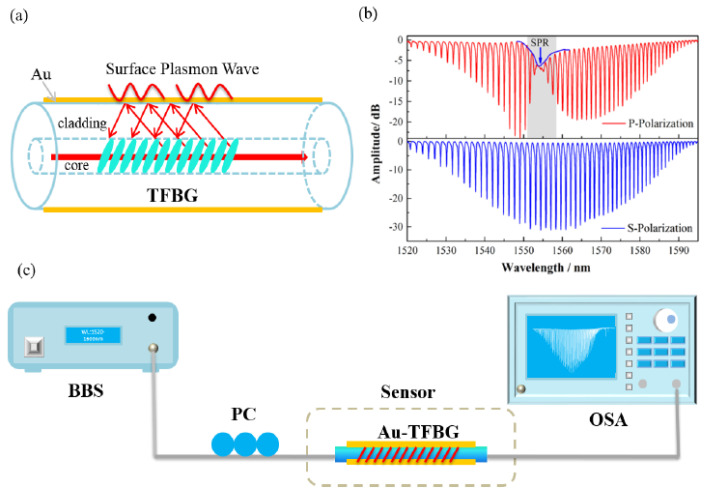
(**a**) Schematic demonstration of tilted fiber Bragg grating plasmonic biosensor in [50]. (**b**) Transmission spectra of the Bragg grating under P and S polarization, and (**c**) experimental setup for detection of bio-species using tilted fiber Bragg grating plasmonic biosensor. Figure reproduced under the terms of the CC-BY Creative Commons attribution 4.0 from Reference [50], Copyright 2017.

Zhang et al. [50] experimentally reported a tilted fiber Bragg grating-based SPR label-free biosensor, which utilize a special boronic acid derivative to detect glycoprotein. Tilted fiber Bragg gratings were inscribed in a conventional SMF-28 fiber using a 248 nm UV excimer laser and the phase-mask method [50]. The Au nanofilm was then deposited around the fiber surface using an ion sputtering instrument, as demonstrated in Figure 21a [50]. The Bragg grating spectrum for p-polarization and s-polarization light illumination are also demonstrated in Figure 21b. The fiber is then illuminated by a broadband source in a 1510–1610 nm wavelength range, and the transmission spectrum was monitored by an optical spectrum analyzer, as demonstrated in Figure 21c [50].

**Figure 22:**
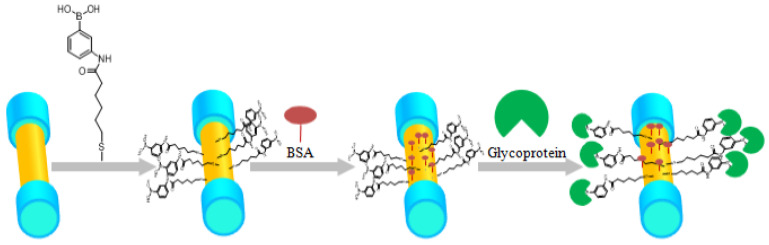
The process of protein identification and immobilization on the plasmonic sensing region of the biosensor for glycoprotein detection. The sensor was first dipped in boronic acid derivative for 24 h, and then block the unreacted carboxyl by dipping the sensor to the BSA, and different concentrations of glycoprotein. Figure reproduced under the terms of the CC-BY Creative Commons attribution 4.0 from Reference [50], Copyright 2017.

The protein identification and immobilization process for boronic acid derivative and glycoprotein in their proposed biosensor is illustrated in Figure 22.

After rinsing the Au-coated plasmonic biosensor probe with ethanol and deionized water, the optical fiber probe was dipped in boronic acid derivative, which was dissolved in the ethanol for 24 h so that the molecules were self-assembled onto the sensor surface [50]. The PBS buffer was firstly pumped into the fiber surface using micro-fluidic channel with specific flow rates [50], and then BSA (bovine serum albumin, 1 mg/mL) flowed over the surface to block the unbounded binging sites on the gold surface [50]. The reporter molecule used in their experiments was (3-((11-mercaptoun-decanamido) methyl) phenyl) boronic acid (PBA with long chain) [50]. The glycoprotein can then bind with PBA and the detection experiments were conducted for different conditions [50]. The proposed plasmonic fiber-optic biosensor exhibits excellent repeatability with a protein concentration sensitivity up to 2.867 dB/(mg/mL) and a limit of detection of 15.56 nM [50].

In Table 1, we summarized different types of fiber-optic biosensors, their main plasmonic material, the target substance, the sensor detection range, and the reported sensitivities/resolutions or limit of detections, which have been reviewed in this review article. It should be noted that while there are lots of other fiber-optic biosensors in the literature, in this paper we only focused on the most recent and the most significant advances in the design of biosensors using plasmonic optical fibers.

## 4. Conclusions and Outlook

The field of plasmonic fiber-optic sensors has grown significantly during the last few years, with a tsunami of publications on different designs, materials, setups, and applications. Combining specialty optical fibers with plasmonics opened the door to new possibilities in biosensing in terms of design principles and detection limits. In comparison, plasmonic fiber-optic biosensors based on conventional optical fibers are usually simple in design and more cost-effective than the biosensors based on specialty optical fibers. However, the flexibility in the design that specialty optical fibers can offer, combined with the potential for utilizing alternative materials, can result in higher sensitivity, limit of detection, and detection range in biosensors based on specialty optical fibers. Between specialty optical fiber based plasmonic biosensors, PCF-based biosensors (in particular D-shaped PCF fibers) are the most studied design concept with lots of potential for future optimization in different biosensing applications. While solid core PCFs explored extensively as the sensing platform for plasmonic sensors, hollow-core PCFs filled with liquid analytes, and alternative designs such as H-shaped PCFs or trapezoid-based PCFs can be potential new candidates for next-generation of plasmonic fiber-optic biosensors.

Furthermore, the use of alternative plasmonic materials can be the main future research direction in the field of plasmonic fiber-optic sensors. Most of the reported designs utilized Au or Ag nanoparticles/nanofilms [22]. However, Au and Ag nanomaterials suffer from high production costs and low melting points, which can significantly limit the large-scale application of these plasmonic materials in commercial fiber-optic sensors [21,22]. A few of the reported designs already utilized alternative plasmonic materials such as graphene [23], graphene oxide [51], titanium nitride [21], titanium oxide [24], Molybdenum trioxides [52,53], zinc oxide [54], platinum based nanocomposites [55], bi layers of indium tin oxide and platinum [56], and tungsten oxide [57] as either the coating materials or the main plasmonic layer. However, further investigations on the performance of these fiber-optic biosensors and comparison between alternative plasmonic materials and Au/Ag nanofilms/NPs are necessary.

Another important area of research within the field of plasmonic fiber-optic sensors can be developing new experimental setups for different fiber-optic designs. The use of novel light sources such as partially coherent beams [58], use of mid-infrared and THz regime light sources and detectors combined with plasmonic materials such as graphene [59,60], and finding cost effective ways to detect a change in intensity of light or location of absorption maxima can potentially transform the field.

Finally, scientists should look ahead to find more applications for plasmonic fiber-optic biosensors in different fields. While these sensors are already taken into practice in some fields such as biomedicine, the vast majority of potential applications remained uncharted. Plasmonic fiber-optic biosensors can easily replace conventional plasmonic biosensors in environmental monitoring, oil industry, ocean monitoring, chemistry, drug discovery, medicine, biology, and industrial research.

## Figures and Tables

**Table 1:** Comparison between the design, materials, and performance of different fiber-optic plasmonic biosensors reported in this review article. Note that LOD is limit of detection, Trap. is an acronym for trapezoid, Circ. is an acronym for circular, MS. is an acronym for micro-structured fiber, and Ab. is an acronym for antibody. The detection range values are expressed in terms of RI of analyte unless specified otherwise.

Reference	Design Category	Plasmonic Material	Target Substance	Detection Range	Max Sensitivity or LOD or Resolution
[26]	Unclad	Ag	Triacylglycerides	0–7 mM	28.5 nm/mM
[27]	Unclad	Ag/ITO	CrO_4_ in solutions	10^−11^–10^−3^ M	0.5 × 10^−11^ M
[28]	Unclad	Au	CR protein	0.01–20 μg/mL	2659.64 nm/RIU
[29]	D-shaped	Au	BSA	1.333–1.404	1.17 nm/(mg/mL)
[30]	D-shaped	Ag	Liquid analytes	1.333–1.345	2166 nm/RIU
[31]	D-shaped	Graphene/Ag	Liquid analytes	1.30–1.36	5161 nm/RIU
[32]	Tapered	Au	Anti-DNP Ab.	10^−9^–10^−6^ g/mL	3.2 × 10^−5^ RIU
[34]	U-shaped	Ag	Mercury ions	2–200 ppb	2 ppb
[35]	U-shaped	Au	*E. coli* bacteria	1.33–1.38	1.5 × 10^3^ CFU/mL
[40]	PCF D-type	Au	Liquid analytes	1.36–1.38	3340 nm/RIU
[41]	PCF D-type	Au	Liquid analytes	1.36–1.41	14,660 nm/RIU
[37]	PCF D-type	Au	Liquid analytes	1.45–1.6	9300 nm/RIU
[21]	PCF D-type	TiN	Liquid analytes	1.44–1.52	−16,275 nm/RIU
[42]	PCF Trap.	Au	Liquid analytes	1.40–1.57	17,000 nm/RIU
[43]	PCF Circ.	Au	Biolayer thickness	-	0.039 nm
[44]	PCF H-type	Au	Liquid analytes	1.33–1.49	25,900 nm/RIU
[45]	MS. H-type	Au/TiO_2_	Liquid analytes	1.32–1.33	5000 nm/RIU
[49]	TBFG	Au	HER2	10^−12^–10^−6^ g/mL	124.89 nm/RIU
[50]	TBFG	Au	Glycoprotein	1.320–1.360	15.56 nM
